# Identifying viral parameters from *in vitro* cell cultures

**DOI:** 10.3389/fmicb.2012.00319

**Published:** 2012-09-04

**Authors:** Shingo Iwami, Kei Sato, Rob J. De Boer, Kazuyuki Aihara, Tomoyuki Miura, Yoshio Koyanagi

**Affiliations:** ^1^Faculty of Sciences, Department of Biology, Kyushu UniversityHigashi-ku, Fukuoka, Japan; ^2^Precursory Research for Embryonic Science and Technology, Japan Science and Technology AgencyKawaguchi, Saitama, Japan; ^3^Institute for Virus Research, Kyoto UniversityKyoto, Japan; ^4^Theoretical Biology and Bioinformatics, Utrecht UniversityUtrecht, Netherlands; ^5^Institute of Industrial Science, The University of TokyoMeguro-ku, Tokyo, Japan; ^6^Graduate School of Information Science and Technology, The University of TokyoMeguro-ku, Tokyo, Japan

**Keywords:** virus infection, mathematical modeling, *in vitro* experiment, quantification

## Abstract

Current *in vitro* cell culture studies of viral replication deliver detailed time courses of several virological variables, like the amount of virions and the number of target cells, measured over several days of the experiment. Each of these time points solely provides a snap-shot of the virus infection kinetics and is brought about by the complex interplay of target cell infection, and viral production and cell death. It remains a challenge to interpret these data quantitatively and to reveal the kinetics of these underlying processes to understand how the viral infection depends on these kinetic properties. In order to decompose the kinetics of virus infection, we introduce a method to “quantitatively” describe the virus infection in *in vitro* cell cultures, and discuss the potential of the mathematical based analyses for experimental virology.

## Introduction

The recent rapid development of experimental techniques in molecular biology and cell biology has revealed many new insights into the complexed interactions between viruses and their target cells. Most of these studies are of a qualitative nature and describe the cellular and molecular details of the interactions. To learn more about the quantitative features of virus replication, we can now generate time courses tracking the dynamics of viruses and target cells in experiments. Each of the time points during a series of experiment provides a snap-shot of the number of target cells, the number of infected cells, and the amount of virions in the culture. The whole time course reflects the results of a complex process consisting of consecutive interactions between viruses, their target cells, infected cells, and viral production. It is difficult to translate these data quantitatively into the parameters identifying the multi-composed kinetics of viral infection. To decompose and quantify the kinetics of virus infection, it will be an extremely useful to rely on mathematical modeling, mathematical analysis, and numerical simulation of the experimental data (Ho et al., 1995; Perelson et al., 1997). Modeling the whole time courses mathematically, we can estimate several parameters underlying the kinetics of virus infection (e.g., the burst size and the basic reproductive number). These parameters cannot be obtained directly by experiments only. Comparing the parameter values between viruses allows one to identify the major functional differences between viruses, and to understand why one is more virulent than the other, and why their time courses are so different. This approach is particularly useful for analyzing data from *in vitro* experiments using cell cultures, because we can nowadays obtain frequent samples of several kinetic variables in a relatively simple environment (as compared to an *in vivo* infection). Indeed, it is now possible to fully parameterize our mathematical models on such *in vitro* data, and to realize quite robust quantification of the virus infection kinetics (Mohler et al., 2005; Beauchemin et al., 2008; Iwami et al., 2012).

The importance and significance of modeling work is slowly becoming recognized in the community of experimental virologists. Starting with the landmark papers revealing the turnover of HIV-1 infected cells *in vivo* from the decline in the viral load in patients following initiation of antiretroviral therapy (Ho et al., 1995; Wei et al., 1995), mathematical modeling has evolved into an important tool in modern biology (Perelson, 2001). Here we introduce our recently developed approach to “quantitatively” describe the kinetics of virus infection in cell cultures employing the full time-course of the data. And we will discuss the potential of such approaches combining experimental and mathematical analyses to address unsolved question in virology by identifying viral parameters.

## Materials and methods

*In vitro* cell culture experimental data on the infection of HSC-F cells with SHIV-KS661 were collected over time courses of 10 consecutive days. Each day most of virus (85.4%), and a small percentage of the cells (5.5%) was removed from the culture supernatant for measurement, and fresh medium was added. The measurement consisted of the concentrations of HSC-F cells positive or negative for a viral antigen, Nef, [cells/ml], and the SHIV-KS661viral load [RNA copies/ml] (Table 1). The experiment was repeated for two different values of the initial viral inoculum (MOI: multiplicity of infection). The time courses were analyzed with the model described below.

**Table 1:** **Experimental data for the *in vitro* experiment**.

**MOI**	**Measurement day**									
	**0**	**1**	**2**	**3**	**4**	**5**	**6**	**7**	**8**	**9**
**CONCENTRATION OF Nef-NEGATIVE HSC-F CELLS (cells/ml)**										
2 × 10^−4^	6400000	6570000	6240000	4795608	4826259	1234110	463638	156560	40843	16200
2 × 10^−5^	6400000	7300000	7690000	5790000	5233650	6005620	2404116	575240	231420	123641
**CONCENTRATION OF Nef-POSITIVE HSC-F CELLS (cells/ml)**										
2 × 10^−4^	d.l.	d.l.	d.l.	15392	483741	1865890	866362	223440	69157	13800
2 × 10^−5^	d.l.	d.l.	d.l.	d.l.	36350	424380	3315884	1394760	468580	46359
**TOTAL VIRAL LOAD OF SHIV-KS661 (RNA copies/ml)**										
2 × 10^−4^	150096	2110000	12000000	322000000	7090000000	26000000000	23400000000	8430000000	1560000000	511000000
2 × 10^−5^	16439	160814	621353	17700000	362000000	2180000000	21600000000	21300000000	9000000000	2360000000

d.l., designates samples in which the concentration was below the detection limit.

### Virus infection

The virus solution of SHIV-KS661 (Shinohara et al., 1999) was prepared in a CD4^+^ human T lymphoid cell line, M8166 (a subclone of C8166) (Clapham et al., 1987), and was stored in liquid nitrogen until use. The HSC-F cell line (Akari et al., 1996) was cultured in a culture medium (RPMI-1640 supplemented with 10% fetal calf serum) at 37°C and 5% CO_2_ in humidified conditions. Each experiment was performed using 2 wells of a 24-well plate with a total suspension volume of 2 ml (1 ml per well) and an initial cell concentration of *T*_0_ = 6.46 × 10^6^ cells/ml in each well. Because the initial cell concentration is close to the carrying capacity of 24-well plates, and HSC-F cells replicate slowly, in the absence of SHIV-KS661 infection, the population of target cells, changes very little on the timescale of our experiment (data not shown). We therefore neglected the effects of potential regeneration of HSC-F cells when constructing the mathematical model. For virus infection, cultures of HSC-F cells were inoculated with two different MOIs [MOI 2.0 × 10^−4^ and MOI 2.0 × 10^−5^, where a MOI of 1 means one 50% tissue culture infectious dose (TCID_50_) per cell] of SHIV-KS661, and were incubated at 37°C. Four hours after inoculation, the cells were washed to remove the remaining viruses and were replaced into a fresh culture medium. The culture supernatant was harvested daily for 10 days, and was replaced with fresh medium. On a daily basis 5.5% of the cells in the culture were harvested to measure the number of target cells and infected cells. Cells were counted by staining them with an anti-SIV Nef monoclonal antibody (04–001, Santa Cruz Biotechnology, Santa Cruz, CA) labeled by Zenon Alexa Fluor 488 (Invitrogen, Carlsbad, CA), as previously described (Iwami et al., 2012). Each harvested supernatant, including 85.4% of the culture virus was stored at −80°C, and the amount of viral RNA was quantified by RT-PCR, as previously described (Iwami et al., 2012).

### Mathematical modeling

**Figure 1:**
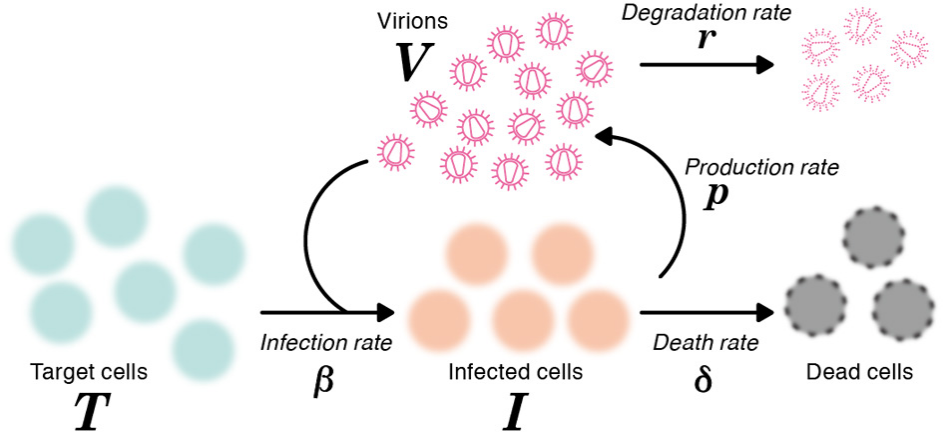
**A schematic representation of the mathematical model**. The variables *T* and *I* are the number of target and infected cells, respectively, and *V* is the number of RNA copies of virus. The parameters δ, *c*, β and *p* represent the death rate of infected cells, the degradation rate of viral RNA, the rate constant for infection of target cells by virus, and the viral production rate of an infected cell, respectively.

To describe the *in vitro* kinetics of virus infection, we used a classical mathematical model that is used widely for analyzing viral kinetics (Perelson and Nelson, 1999; Nowak and May, 2000; Perelson, 2001):

(1) $$\frac{dT}{dt}=-\beta TV,\;\frac{dT}{dt}=\beta TV\;-\;\delta I,\;\frac{dV}{dt}=\;pI\;-\;cV,$$

where *T* and *I* are the numbers of target (susceptible) cells, and infected (virus-producing) cells per ml of medium, respectively, and *V* is the number of RNA copies of virus per ml of medium. The parameters δ, *c*, β and *p* represent the death rate of infected cells, the degradation rate of viral RNA, the rate constant for infection of target cells by virus, and the viral production rate of an infected cell, respectively. The basic model (1) is a simplified version of the previously developed mathematical model in (Iwami et al., 2012), because we here use the time-course data including only viral RNA (but not both viral RNA and infectivity). A schematic of the basic model is shown in Figure 1.

### Data fitting

Due to the daily harvesting of cells and virus, in our model the concentrations of target and infected cells must be reduced by 5.5% per day, and the viral loads (RNA copies) have to be reduced by 85.4% per day. We approximate these losses by adding continuous exponential decay terms, −*dT*, −*dI*, and −*rV*, to the equations, respectively, where *d* = 0.057 per day to account for the harvesting of cells, and *r* = 1.93 per day for the collection of virus. The degradation rate of virus was estimated to be *c* = 0.039 per day in separate experiments (data not shown). The remaining three parameters (δ, β, *p*), along with the 6 initial (*t* = 0) values for the variables (three for each of the two MOI values), were determined by fitting Equation (1) to the data. We simultaneously fit Equation (1) to the concentrations of Nef-negative and Nef-positive HSC-F cells and the viral loads for both MOIs, using nonlinear least-squares regression [using the FindMinimum package of *Mathematica8.0* that minimizes the sum of squared residuals (SSR)]. Experimental measurements below the detection limit were excluded when computing the SSR.

## Results and discussion

**Figure 2:**
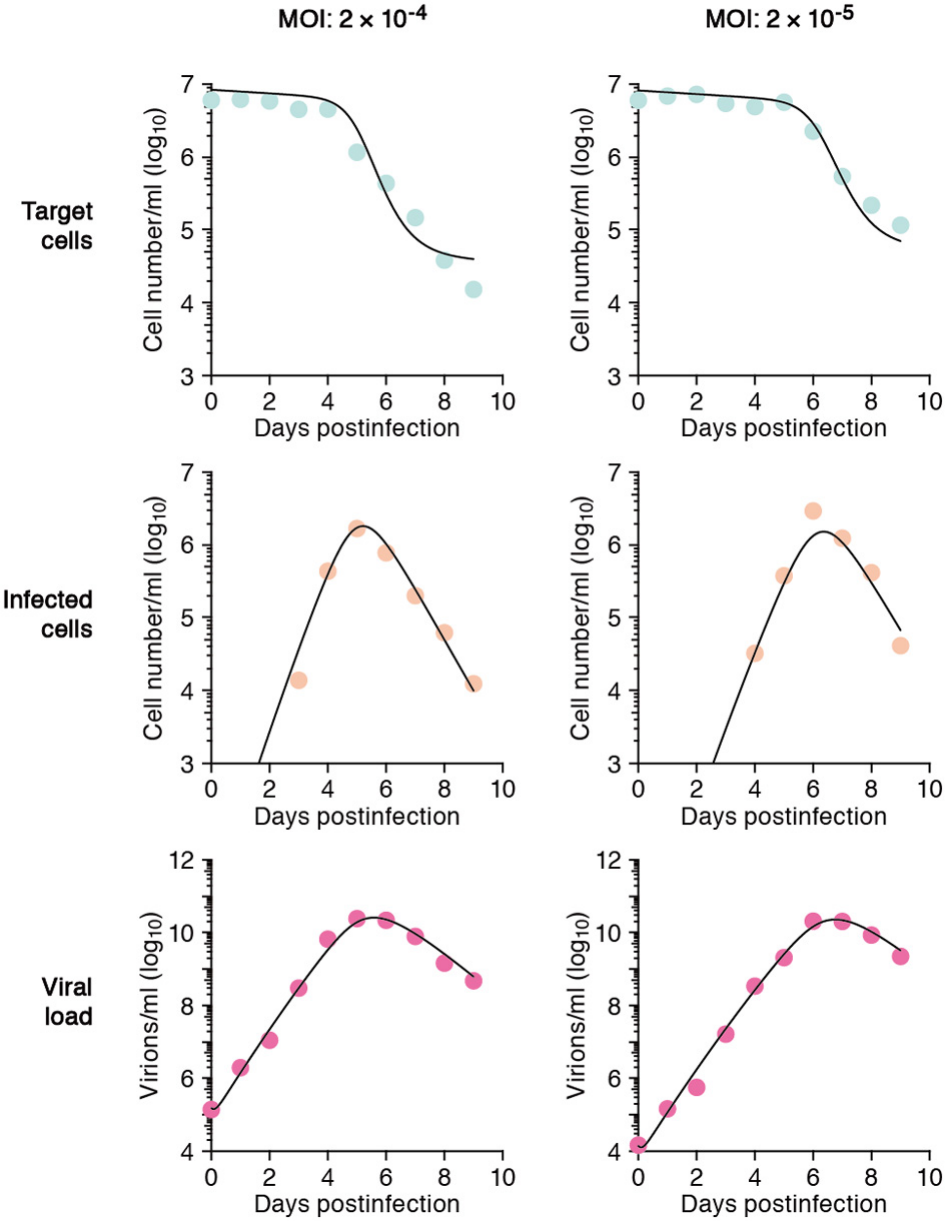
**The mathematical model describes the data well**. HSC-F cells were inoculated with SHIV-KS661 24 h before *t* = 0 and each *in vitro* experimental quantity was measured daily from *t* = 0 d to 9 *d*. The curves depict the best fit of the model (Equation 1), to the experimental data of SHIV-KS661 infection *in vitro* (symbols) for the target cells, infected cells, and the viral load for the two different experiments conducted at different MOIs. All data were fitted simultaneously as described in “Materials and Methods.”

In total we obtained 53 data points for quantifying the kinetics of SHIV-KS661 *in vitro* cell cultures. Using a previously established estimate for the degradation rates of RNA (*c*) *in vitro* culture, we estimated the values of the three remaining unknown parameters (δ, β, *p*) and the six initial values. The parameter estimates obtained by fitting Equation (1) to the full *in vitro* dataset simultaneously as described in “Materials and Methods” are given in Tables 2 and 3. These estimates are similar to our previous parameter estimates in (Iwami et al., 2012). The behavior of the model using these best-fit parameter estimates is shown together with the data in Figure 2, which reveals that the relatively simple model of Equation (1) describes these *in vitro* data very well. This suggests that the parameters that were estimated are representative for the various processes underlying the viral kinetics. Let us discuss what we can learn from these data.

**Table 2:** **Parameters values and derived quantities**.

**Parameter Name**	**Symbol**	**Unit**	**Value**
**PARAMETERS OBTAINED FROM SIMULTANEOUS FIT TO FULL *in vitro* DATASET**			
Rate constant for infection	β	(RNA/ml · day)^−1^	8.61 × 10^−11^
Death rate of infected cells	δ	day^−1^	1.75
Production rate of total virus	*p*	RNA copies · day^−1^	3.26 × 10^4^
**QUANTITIES DERIVED FROM FITTED VALUES**			
Half-life of infected cells	log 2/δ	days	0.40
Viral burst size	*p*/δ	RNA copies	1.87 × 10^4^
Basic reproductive number of virus	*R*_0_	–	5.10

**Table 3:** **Fitted initial values for the *in vitro* experiment**.

**Variable**	**Unit**	**Fitted initial value at MOI of**	
		**2 × 10^−4^**	**2 × 10^−5^**
*T(0)*	cells/ml	8.36 × 10^6^	8.18 × 10^6^
*I(0)*	cells/ml	1.13	3.45 × 10^−4^
*V(0)*	RNA copies/ml	1.50 × 10^5^	1.41 × 10^4^

### Half-life of infected cells (log 2/δ)

The death rate of infected cells was estimated to be δ = 1.75 per day. Since in differential equations the time to death is exponentially distributed (Holder and Beauchemin, 2011), this death rate corresponds to a half-life of log 2/δ = 0.40*d*, and an average life-span of 1/δ = 0.57 *d*, of productively infected HSC-F cells. Because the Nef protein is primarily produced at a late phase of the viral replication in a cell, and since we do not distinguish between an early eclipse phase and a late phase of virus production in our model, the “infected cells” that our model describes should largely correspond to cells at a relatively late stage of infection (Iwami et al., 2012). The half-life that we estimate should therefore apply primarily for infected cells at a late stage of infection, and need not apply for cells in the early eclipse phase.

### Burst size (*p*/δ)

**Figure 3:**
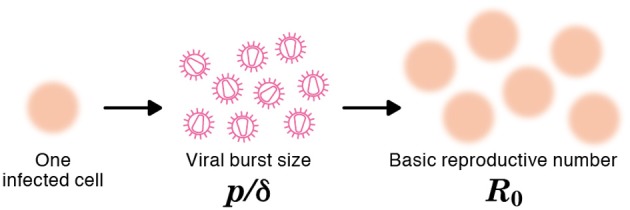
**The burst size and the basic reproductive number of a virus**. The burst size is defined as the expected number of virions produced by one infected cell over its life-time (e.g., *p*/δ = 9 in the figure). The basic reproductive number, *R*_0_, is defined as the expected number of newly infected cells resulting from one infected cell during its life-time (e.g., *R*_0_ = 6 in the figure).

The viral production rate of an infected cell was estimated to be *p* = 3.26 × 10^4^ RNA copies per day. Because an infected cell in the model produces virus over an average of 1/δ days, the total viral burst size can be estimated as *p*/δ = 1.87 × 10^4^ RNA copies per cell. This *in vitro* estimate is in reasonable agreement with recent *in vivo* estimates obtained using single-cycle SIV (Chen et al., 2007). The total burst size is defined as the total number of virions produced by any one infected cell during its life-time (Nowak and May, 2000; Beauchemin et al., 2008; Iwami et al., 2012) (see Figure 3), and is often considered as a normalized viral replication property reflecting the trade-off between viral production (*p*) and its cytopathic effects (δ).

### Basic reproductive number (*R*_0_)

The average number of newly infected cells produced from any one infected cell, under conditions where the most of the target cells are uninfected, is known as the basic reproductive number *R*_0_, and is an important parameter predicting the course of infection (Nowak et al., 1997; Nowak and May, 2000; Ribeiro et al., 2010). Any one infected cell produces a progeny of *p*/(δ + *d*) viruses before the cell dies, or is removed from the culture, and each produced virus will infect target cells at a constant rate β, until the virus is cleared or harvested (i.e., over 1/(*r* + *c*) days on average). At the beginning of the experiment there are *T*_0_ target cells. Thus, using our parameter estimates, the reproductive number is calculated as *R*_0_ = β*pT*_0_/[(δ + *d*)(*r* + *c*)] = 5.10 in *in vitro* culture experiments (Nowak and May, 2000; Beauchemin et al., 2008; Iwami et al., 2012) (see Figure 3). The basic reproductive number characterizes the course of the infection in cell culture. For example, one can predict the fraction of target cells that will be removed by the infection through the recursive relation 1 − *f*_*I*_ = e^−*R*_0_*f*_*I*_^, which is called the “final size equation” (Anderson, 1991; Iwami et al., 2012). Here the parameter *f*_*I*_ corresponds to the fraction of target cells that are eventually removed by the infection (i.e., *f*_*I*_ = 1 − *T*(∞)/*T*_0_). Using the *R*_0_ = 5.1 we find that *f*_*I*_ = 0.9937, and that the fraction of surviving target cells at the end of the infection should approach 1-*f*_*I*_ = 0.0063. In our experiments this implies a final target cell population of approximately *T* (∞) = 4.03 × 10^4^ cells/ml. This value agrees well with the final size of Nef-negative HSC-F cells in the MOI 2.0 × 10^−4^ experiment, where *T* (9) = 1.62 × 10^4^ cells/ml. Thus, the basic reproductive number provides valuable information about the expected course of infection. Note that at the MOI of 2.0 × 10^−5^ the infection is so slow that the final target cell value has not yet been approached at day 9 (see Figure 2).

## Conclusion

Combining mathematical modeling with experimental data, we have been able to estimate several parameters defining the kinetics of SHIV-KS661 infecting HSC-F cells, from just two time courses of an *in vitro* infection. For this it was essential that we had the full time-course of the infection available for fitting the model. The data before the peak of virus infection, i.e., the up-slope of the number of viral RNA copies in the culture supernatant, reflects virus production, while the data after the peak, i.e., the down-slopes of the viral load and the infected cells, reflect the death of infected cells and viral clearance. Thus to reliably estimate the kinetic parameters, one needs to collect time-course data throughout the infection.

## Perspective

Our results of an SHIV infection in an *in vitro* cell culture are a simple example of a quantitative analysis of virus infection dynamics employing on mathematical and computational methods. Our approach can be applied regardless of viral family and genus. To further explore how our approach of modeling time-course data can be used in future work we will discuss a number of hypothetical examples, emphasizing how quantitative estimates can be used to address unsolved question in virology.

### Identifying the major differences among several viral strains

**Figure 4:**
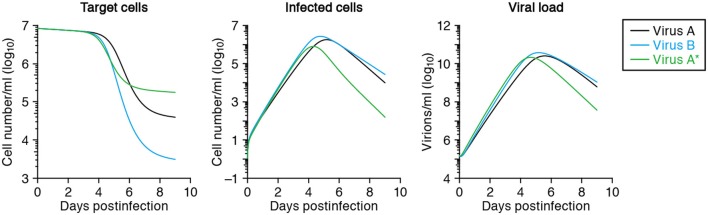
**Predicted virus infection kinetics with different parameters**. The solid curves show the predicted kinetics of target cells (left), infected cells (middle) and virus load (right) following infection with SHIV-KS661 at an MOI 2 × 10^−4^ using the parameters in Tables 2 and 3. The time *t* = 0 corresponds to 24 h after inoculation of SHIV-KS661 to HSC-F. In the text we call this virus-A. The dashed and dotted curves show those of the variants virus-B and virus-A^*^, respectively. Virus-B is less cytopathic and has a 1.5-fold decreased death rate of infected cells (δ = 1.16). Cells infected with the more virulent “mutant” virus-A^*^ die 3-fold faster than those infected with the wild-type A (i.e., δ = 5.25 vs. δ = 1.75, respectively), but produce 2-fold more virus (*p* = 6.72 × 10^4^) per day than those infected with “wild-type” virus-A.

After fitting time-course data from different virus strains, one can compare the estimated parameters of each viral strain, such as its half-life of infected cells, burst size and basic reproductive number, to reveal the quantitatively largest differences between the strains (Mitchell et al., 2011). For example, let us denote SHIV-KS661 as “virus-A,” which brings about a half-life of 0.4 days in infected cells, and consider a less cytopathic variant “virus-B” extending the half-life 1.5-fold to 0.6 days. The expected time-courses of these two variants are depicted in Figure 4, and reveal a major difference in the target cell dynamics and minor differences in the number of infected cells and the viral load (compare the solid line with the dashed lines, depicting virus A and B, respectively). If one were to fit the *in silico* data from virus A and B in Figure 4 with our mathematical model, one would correctly conclude that the half-life of infected cells of virus-B is 1.5 times longer than that of cells infected with virus-A, and therefore that virus B is less cytopathic than virus A. It is difficult to arrive at that result by just visual inspection of the data, however. The effect of cytopathicity on the time courses of target cells, infected cells, and virus load are difficult to predict intuitively. Additionally, there is no experimental technique available to measure quantities like the cytopathicity directly as an absolute value. For the production rate, the burst size and the basic reproductive number of the virus, similar arguments apply, and one has to rely on modeling to identify quantitative differences between viral strains.

### Identifying the function of viral proteins or amino acids in infection

Using molecular cell biology techniques, we are currently able to investigate the function of individual viral proteins in several aspects of viral replication. However, it remains difficult to interconnect those particular results and to integrate the roles of different molecules in terms of the overall parameters defining a virus infection, like a replication rate or a burst size. By modeling *in vitro* time courses, and comparing the estimated parameters between a wild-type virus and several particular mutants, one can quantify the role of every amino acid mutation on the several parameters defining a virus infection. For example, if one were to take SHIV-KS661 as a “wild-type virus-A,” with an estimated half-life of infected cells of 0.40 days and a viral production rate of 3.26 × 10^4^ RNA copies per day (see Table 2), and find by fitting that a more virulent mutant “virus-A^*^” has a 3-fold shorter half-life of its infected cells, but a 2.0-fold increased production rate of 6.52 × 10^4^ RNA copies per day, one would be able to conclude that this particular mutation decreases the total viral production per generation to 2/3 of that of the wild-type. Thus, the more virulent virus is less fit, i.e., has a lower *R*_0_, because the total burst sizes of virus-A and virus-A^*^ are 1.87 × 10^4^ and 1.25 × 10^4^ RNA copies per generation, respectively. For the function of the mutated protein one would be able to conclude that it plays a role in the production of novel viral particles, and that increased production apparently brings about a shorter expected life-span of infected cells. The solid and dotted curves in Figure 4 show the virus kinetics predicted by Equation (1) for virus-A (solid line) and virus-A* (dotted line), respectively. Similar approaches allow us to also investigate the functions of multiple mutations in possibly several proteins quantitatively.

### Finding the target of novel antiviral compounds

Calculating and comparing parameter estimates in the absence and presence of an antiviral compound, allows one to investigate the function of the compound in a very similar manner (Baccam et al., 2006; Beauchemin et al., 2008). For instance, if the daily viral production rate is reduced to half but the half-life of infected cells has remained similar, one concludes that the compound inhibits viral production without affecting cytopathicity. In addition, if a dose-dependent basic reproductive number, *R*_o_, were obtained, one would estimate how effectively the compound inhibits total viral replication. From the value of 1−1/*R*_0_ (Anderson, 1991), one can calculate the critical compound concentration at which the infection should die out. Note that this value is not the same as the conventional IC_50_, the half maximal (50%) inhibitory concentration. Identifying the precise mode of action of novel compounds may help the development of novel antiviral drugs.

### Future direction

As discussed above, an approach of combining experiments with mathematical modeling has broad applications in virology. One possible extension of our model is to also consider the “eclipse” phase of the infection of a cell to allow for a period in which no virus is produced and the cell may have a different death rate (Baccam et al., 2006; Beauchemin et al., 2008; Iwami et al., 2012). Another extension is to divide the viral population into infectious and non-infectious virus, because the virus that is produced by most of the cells is non-infectious (Schulze-Horsel et al., 2009; Iwami et al., 2012). In Equation (1), it is assumed all virus is infectious, and the non-infectious fraction is in fact incorporated by a lower infection rate β. If one were to have data on the amounts of infectious and non-infectious virions, and/or on the fraction of infected cells in the eclipse phase, one can extend the mathematical model accordingly and obtain even more detailed quantification of the characteristics of any virus studied in particular culture conditions. Furthermore, it is challenging but very interesting to distinguish cell-free and cell-to-cell infection, which are two different mode of viral infection, and to quantify the efficacy of each mode. Sourisseau et al. reported that in a continuously shaken culture in a HIV replication assay cell-to-cell infection is blocked (Sourisseau et al., 2007). Combining a novel mathematical model including both a cell-free and a cell-to-cell infection mode, and fitting that to shaken and non-shaken HIV replication assays, we might be able to quantitatively identify the two infection modes. Summarizing, our method of modeling time courses of viral infection is effectively capable of describing the data, and therefore provides a new approach of characterizing and comparing viruses in a quantitative manner to better understand their infection kinetics under *in vivo* circumstances.

### Conflict of interest statement

The authors declare that the research was conducted in the absence of any commercial or financial relationships that could be construed as a potential conflict of interest.
